# GRIM-Filter: Fast seed location filtering in DNA read mapping using processing-in-memory technologies

**DOI:** 10.1186/s12864-018-4460-0

**Published:** 2018-05-09

**Authors:** Jeremie S. Kim, Damla Senol Cali, Hongyi Xin, Donghyuk Lee, Saugata Ghose, Mohammed Alser, Hasan Hassan, Oguz Ergin, Can Alkan, Onur Mutlu

**Affiliations:** 1 Department of Electrical and Computer Engineering, Carnegie Mellon University, Pittsburgh, PA USA; 2 Department of Computer Science, Carnegie Mellon University, Pittsburgh, PA USA; 3 NVIDIA Research, Austin, TX USA; 4 Department of Computer Engineering, Bilkent University, Bilkent, Ankara Turkey; 5 Department of Computer Engineering, TOBB University of Economics and Technology, Sogutozu, Ankara Turkey; 6 Department of Computer Science, ETH Zürich, Zürich, CH Switzerland

**Keywords:** High throughput sequencing, Genome sequencing, Seed location filtering, 3D-stacked DRAM, Processing-in-memory, Emerging memory technologies

## Abstract

**Background:**

Seed location filtering is critical in DNA read mapping, a process where billions of DNA fragments (reads) sampled from a donor are mapped onto a reference genome to identify genomic variants of the donor. State-of-the-art read mappers 1) quickly generate possible mapping locations for seeds (i.e., smaller segments) within each read, 2) extract reference sequences at each of the mapping locations, and 3) check similarity between each read and its associated reference sequences with a computationally-expensive algorithm (i.e., sequence alignment) to determine the origin of the read. A seed location filter comes into play before alignment, discarding seed locations that alignment would deem a poor match. The ideal seed location filter would discard all poor match locations prior to alignment such that there is no wasted computation on unnecessary alignments.

**Results:**

We propose a novel seed location filtering algorithm, GRIM-Filter, optimized to exploit 3D-stacked memory systems that integrate computation within a logic layer stacked under memory layers, to perform processing-in-memory (PIM). GRIM-Filter quickly filters seed locations by 1) introducing a new representation of coarse-grained segments of the reference genome, and 2) using massively-parallel in-memory operations to identify read presence within each coarse-grained segment. Our evaluations show that for a sequence alignment error tolerance of 0.05, GRIM-Filter 1) reduces the false negative rate of filtering by 5.59x–6.41x, and 2) provides an end-to-end read mapper speedup of 1.81x–3.65x, compared to a state-of-the-art read mapper employing the best previous seed location filtering algorithm.

**Conclusion:**

GRIM-Filter exploits 3D-stacked memory, which enables the efficient use of processing-in-memory, to overcome the memory bandwidth bottleneck in seed location filtering. We show that GRIM-Filter significantly improves the performance of a state-of-the-art read mapper. GRIM-Filter is a universal seed location filter that can be applied to any read mapper. We hope that our results provide inspiration for new works to design other bioinformatics algorithms that take advantage of emerging technologies and new processing paradigms, such as processing-in-memory using 3D-stacked memory devices.

## Background

Our understanding of human genomes today is affected by the ability of modern technology to quickly and accurately determine an individual’s entire genome. The human genome is comprised of a sequence of approximately 3 billion bases that are grouped into deoxyribonucleic acids (*DNA*), but today’s machines can identify DNA only in short sequences (i.e., *reads*). Determining a genome requires three stages: 1) cutting the genome into many short reads, 2) identifying the DNA sequence of each read, and 3) mapping each read against the reference genome in order to analyze the variations in the sequenced genome. In this paper, we focus on improving the third stage, often referred to as *read mapping*, which is a major computational bottleneck of a modern genome analysis pipeline. Read mapping is performed computationally by *read mappers* after each read has been identified.

**Fig. 1:**
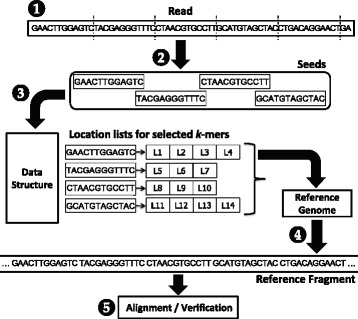
Flowchart of a seed-and-extend mapper

*Seed-and-extend* mappers [1–6] are a class of read mappers that break down each read sequence into *seeds* (i.e., smaller segments) to find locations in the reference genome that closely match the read. Figure 1 illustrates the five steps used by a seed-and-extend mapper. First, the mapper obtains a read (❶ in the figure). Second, the mapper selects smaller DNA segments from the read to serve as seeds (❷). Third, the mapper indexes a data structure with each seed to obtain a list of possible locations within the reference genome that could result in a match (❸). Fourth, for each possible location in the list, the mapper obtains the corresponding DNA sequence from the reference genome (❹). Fifth, the mapper aligns the read sequence to the reference sequence (❺), using an expensive sequence alignment (i.e., verification) algorithm to determine the similarity between the read sequence and the reference sequence.

To improve the performance of seed-and-extend mappers, we can utilize *seed location filters*, recently introduced by Xin et al. [7]. A seed location filter efficiently determines whether a candidate mapping location would result in an incorrect mapping *before* performing the computationally-expensive sequence alignment step for that location. As long as the filter can eliminate possible locations that would result in an incorrect mapping *faster* than the time it takes to perform the alignment, the entire read mapping process can be substantially accelerated [7–10]. As a result, several recent works have focused on optimizing the performance of seed location filters [7–12].

With the advent of seed location filters, the performance bottleneck of DNA read mapping has shifted from sequence alignment to seed location filtering [7–10]. Unfortunately, a seed location filter requires large amounts of memory bandwidth to process and characterize each of the candidate locations. Our goal is to reduce the time spent in filtering and thereby improve the speed of DNA read mapping. To this end, we present a new algorithm, *GRIM-Filter*, to efficiently filter locations with high parallelism. We design GRIM-Filter such that it is well-suited for implementation on 3D-stacked memory, exploiting the parallel and low-latency processing capability in the logic layer of the memory.

3D-stacked DRAM [13–22] is a new technology that integrates logic and memory in a three-dimensional stack of dies with a large internal data transfer bandwidth. This enables the bulk transfer of data from each memory layer to a logic layer that can perform simple parallel operations on the data.

Conventional computing requires the movement of data on the long, slow, and energy-hungry buses between the CPU processing cores and memory such that cores can operate on data. In contrast, processing-in-memory (PIM)-enabled devices such as 3D-stacked memory can perform simple arithmetic operations very close to where the data resides, with high bandwidth and low latency. With carefully designed algorithms for PIM, application performance can often be greatly improved (e.g., as shown in [19–21, 23]) because the relatively narrow and long-latency bus between the CPU cores and memory no longer impedes the speed of computation on the data.

**Our goal** is to develop a seed location filter that exploits the high memory bandwidth and processing-in-memory capabilities of 3D-stacked DRAM to improve the performance of DNA read mappers.

To our knowledge, this is the **first** seed location filtering algorithm that accelerates read mapping by overcoming the memory bottleneck with PIM using 3D-stacked memory technologies. GRIM-Filter can be used with any read mapper. However, in this work we demonstrate the effectiveness of GRIM-Filter with a hash table based mapper, mrFAST with FastHASH [7]. We improve the performance of hash table based read mappers while maintaining their high sensitivity and comprehensiveness (which were originally demonstrated in [3]).

**Key mechanism.** GRIM-Filter provides a quick method for determining whether a read will **not** match at a given location, thus allowing the read mapper to skip the expensive sequence alignment process for that location. GRIM-Filter works by counting the existence of small segments of a read in a genome region. If the count falls under a threshold, indicating that many small segments of a read are *not* present, GRIM-Filter discards the locations in that region before alignment. The existence of all small segments in a region are stored in a bitvector, which can be easily predetermined for each region of a reference genome. The bitvector for a reference genome region is retrieved when a read must be checked for a match in the region. We find that this regional approximation technique not only enables a high performance boost via high parallelism, but also improves filtering accuracy over the state-of-the-art. The filtering accuracy improvement comes from the finer granularity GRIM-Filter uses in counting the subsequences of a read in a region of a genome, compared to the state-of-the-art filter [7].

**Key results.** We evaluate GRIM-Filter qualitatively and quantitatively against the state-of-the-art seed location filter, *FastHASH* [7]. Our results show that GRIM-Filter provides a *5.59x–6.41x* smaller false negative rate (i.e., the proportion of locations that pass the filter, but that truly result in a poor match during sequence alignment) than the best previous filter with *zero* false positives (i.e., the number of locations that do not pass the filter, but that truly result in a good match during sequence alignment). GRIM-Filter provides an end-to-end performance improvement of *1.81x–3.65x* over a state-of-the-art DNA read mapper, *mrFAST* with *FastHASH*, for a set of real genomic reads, when we use a sequence alignment error tolerance of 0.05. We also note that as we increase the sequence alignment error tolerance, the performance improvement of our filter over the state-of-the-art increases. This makes GRIM-Filter more effective and relevant for future-generation error-prone sequencing technologies, such as nanopore sequencing [24, 25].

## Motivation and aim

Mapping reads against a reference genome enables the analysis of the variations in the sequenced genome. As the throughput of read mapping increases, more large-scale genome analyses become possible. The ability to deeply characterize and analyze genomes at a large scale could change medicine from a reactive to a preventative and further personalized practice. In order to motivate our method for improving the performance of read mappers, we pinpoint the performance bottlenecks of modern-day mappers on which we focus our acceleration efforts. We find that across our data set (see “Methods” section), a state-of-the-art read mapper, mrFAST with FastHASH [7], on average, spends 15% of its execution time performing sequence alignment on locations that are found to be a match, and 59% of its execution time performing sequence alignment on locations that are discarded because they are not found to be a match (i.e., *false locations*).

Our *goal* is to implement a *seed location filter* that reduces the wasted computation time spent performing sequence alignment on such false locations. To this end, a seed location filter would quickly determine if a location will *not* match the read and, if so, it would avoid the sequence alignment altogether. The *ideal* seed location filter correctly finds all false locations without increasing the time required to execute read mapping. We find that such an ideal seed location filter would improve the *average* performance of mrFAST (with FastHASH) by *3.2x*. This speedup is primarily due to the reduced number of false location alignments. In contrast, most prior works [26–40] gain their speedups by implementing all or part of the read mapper in specialized hardware or GPUs, focusing mainly on the acceleration of the sequence alignment process, *not* the *avoidance* of sequence alignment. These works that accelerate sequence alignment provide orthogonal solutions, and could be implemented together with seed location filters, including GRIM-Filter, for additional performance improvement (see “Related work” section for more detail).

## GRIM-Filter

We now describe our proposal for a new seed location filter, GRIM-Filter. At a high level, the key idea of GRIM-Filter is to store and utilize metadata on *short segments* of the genome, i.e., segments on the order of several hundred base pairs long, in order to quickly determine if a read will **not** result in a match at that genome segment.

### Genome metadata representation

**Fig. 2:**
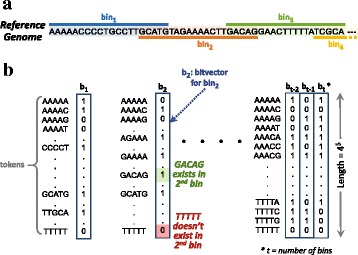
GRIM-Filter has a 2D data structure where each bit at <row, column > indicates if a token (indexed by the row) is present in the corresponding bin (indicated by the column). **a** GRIM-Filter divides a genome into overlapping bins. **b** GRIM-Filter’s metadata associated with a reference genome. Columns are indexed by the bin number of each location. Rows are indexed by the token value. In this figure, token size=5

Figure 2 shows a reference genome with its associated metadata that is formatted for efficient operation by GRIM-Filter. The reference genome is divided into short contiguous segments, on the order of several hundreds of base pairs, which we refer to as *bins*. GRIM-Filter operates at the granularity of these bins, performing analyses on the metadata associated with each bin. This metadata is represented as a *bitvector* that stores whether or not a *token*, i.e., a short DNA sequence on the order of 5 base pairs, is present within the associated bin. We refer to each bit in the bitvector as an *existence bit*. To account for all possible tokens of length *n*, each bitvector must be 4^*n*^ bits in length, where each bit denotes the existence of a particular token instance. Figure 2 highlights the bits of two token instances of *bin*_2_’s bitvector: it shows that 1) the token GACAG (green) exists in *bin*_2_, i.e., the existence bit associated with the token GACAG is set to 1 in the *b*_2_ bitvector; and 2) the token TTTTT (red) is not present in *bin*_2_, i.e., the existence bit associated with the token TTTTT is set to 0 in the *b*_2_ bitvector.

Because these bitvectors are associated with the reference genome, the bitvectors need to be generated only once per reference, and they can be used to map any number of reads from other individuals of the same species. In order to generate the bitvectors, the genome must be sequentially scanned for every possible token of length *n*, where *n* is the selected token size. If *bin*_*x*_ contains the token, the bit in the *b*_*x*_ bitvector corresponding to the token must be set (1). If *bin*_*x*_ does not contain the token, then the same bit is left unset (i.e., 0). These bitvectors are saved and stored for later use when mapping reads to the same reference genome, i.e., they are part of the genome’s metadata.

### GRIM-Filter operation

Before sequence alignment, GRIM-Filter checks each bin to see if the bin contains a potential mapping location for the read, based on the list of potential locations provided by the read mapper. If the bin contains a location, GRIM-Filter then checks the bin to see if the location is likely to match the read sequence, by operating on the bitvector of the bin.

This relies on the *entire read* being contained within a given bin, and thus requires the bins to overlap with each other in the construction of the metadata (i.e., some base pairs are contained in multiple bins), as shown in Fig. 2a.

GRIM-Filter uses the described bitvectors to *quickly* determine whether a match within a given error tolerance is impossible. This is done before running the expensive sequence alignment algorithm, in order to reduce the number of unnecessary sequence alignment operations. For each location associated with a seed, GRIM-Filter 1) loads the bitvector of the bin containing the location; 2) operates on the bitvector (as we will describe shortly) to quickly determine if there will be no match (i.e., a poor match, given the error tolerance threshold); and 3) discards the location if it determines a poor match. If GRIM-Filter does **not** discard the location, the sequence at that location **must** be aligned with the read to determine the match similarity.

**Fig. 3:**
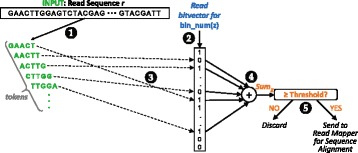
Flow diagram for our seed location filtering algorithm. GRIM-Filter takes in a read sequence and sums the existence of its tokens within a bin to determine whether 1) the read sequence must be sequence aligned to the reference sequence in the bin or 2) it can be discarded without alignment. Note that token size = 5 in this example

Using the circled steps in Fig. 3, we explain in detail how GRIM-Filter determines whether to discard a location *z* for a read sequence *r*. We use *bin_num*(*z*) to indicate the number of the bin that contains location *z*.

GRIM-Filter extracts every token contained within the read sequence *r* (❶ in the figure). Then, GRIM-Filter loads the bitvector for ${bin}_{bin\_num(z)}$ (❷). For each of the tokens contained in *r*, GRIM-Filter extracts the existence bit of that token from the bitvector (❸), to see whether the token exists somewhere within the bin. GRIM-Filter sums all of the extracted existence bits together (❹), which we refer to as the *accumulation sum* for location *z* (*Sum*_*z*_). The accumulation sum represents the number of tokens from read sequence *r* that are present in ${bin}_{bin\_num(z)}$. A larger accumulation sum indicates that more tokens from *r* are present in the bin, and therefore the location is more likely to contain a match for *r*. Finally, GRIM-Filter compares *Sum*_*z*_ with a constant *accumulation sum threshold* value (❺), to determine whether location *z* is likely to match read sequence *r*. If *Sum*_*z*_ is greater than or equal to the threshold, then *z* is likely to match *r*, and the read mapper must perform sequence alignment on *r* to the reference sequence at location *z*. If *Sum*_*z*_ is less than the threshold, then *zwill not* match *r*, and the read mapper skips sequence alignment for the location. We explain how we determine the accumulation sum threshold in “Determining the accumulation sum threshold” section.

Once GRIM-Filter finishes checking each location, it returns control to the read mapper, which performs sequence alignment on *only* those locations that pass the filter. This process is repeated for all seed locations, and it significantly reduces the number of alignment operations, ultimately reducing the end-to-end read mapping runtime (as we show in “Results” section). Our implementation of GRIM-Filter ensures a zero *false positive rate* (i.e., no locations that result in correct mappings for the read sequence are incorrectly rejected by the filter), as GRIM-Filter passes any seed location whose bin contains enough of the same tokens as the read sequence.

GRIM-Filter can also account for errors in the sequence, when some of the tokens do not match perfectly (see “Taking errors into account” section). Therefore, using GRIM-Filter to filter out seed locations does *not* affect the correctness of the read mapper.

### Integration with a full read mapper

**Fig. 4:**
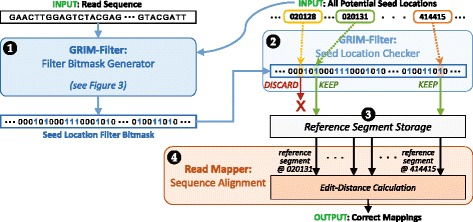
GRIM-Filter integration with a read mapper. The *Filter Bitmask Generator* uses the bitvectors for each bin to determine whether any locations within the bin are potential matches with the read sequence, and saves potential match information into a *Seed Location Filter Bitmask*. The *Seed Location Checker* uses the bitmask to retrieve the corresponding reference segments for only those seed locations that match, which are then sent to the read mapper for sequence alignment

Figure 4 shows how we integrate GRIM-Filter with a read mapper to improve read mapping performance. Before the read mapper begins sequence alignment, it sends the read sequence, along with all potential seed locations found in the hash table for the sequence, to GRIM-Filter. Then, the *Filter Bitmask Generator* for GRIM-Filter performs the seed location filtering algorithm we describe in *GRIM-Filter Operation*, checking only the bins that include a potential seed location to see if the bin contains the same tokens as the read sequence (❶ in Fig. 4). For each location, we save the output of our threshold decision (the computation of which was shown in Fig. 3) as a bit within a *seed location filter bitmask*, where a 1 means that the location’s accumulation sum was greater than or equal to the threshold, and a 0 means that the accumulation sum was less than the threshold. This bitmask is then passed to the *Seed Location Checker* (❷ in Fig. 4), which locates the reference segment corresponding to each seed location that passed the filter (❸) and sends the reference segment to the read mapper. The read mapper then performs sequence alignment on *only* the reference segments it receives from the seed location checker (❹), and outputs the correct mappings for the read sequence.

### Determining the accumulation sum threshold

We now discuss in detail how to determine the threshold used to evaluate the accumulation sum (*Sum*_*z*_). The threshold is used to determine whether or not a seed location should be sent to the read mapper for sequence alignment (shown as ❺ in Fig. 3). A greater value of *Sum*_*z*_ indicates that the seed location *z* is more likely to be a good match for the read sequence *r*. However, there are cases where *Sum*_*z*_ is high, but the read sequence results in a poor match with the seed location *z*. A simple example of this poor match is a read sequence that consists entirely of “A” base pairs, resulting in 100 AAAAA tokens, and a seed location that consists entirely of “G” base pairs except for a single AAAAA token. In this example, all 100 AAAAA tokens in the read sequence locate the one AAAAA token in the seed location, resulting in an accumulation sum of 100, even though the location contains only one AAAAA token. Because such cases occur, even though they may occur with low probability, GRIM-Filter cannot **guarantee** that a high accumulation sum for a seed location corresponds to a good match with a read sequence. On the other hand, GRIM-Filter can guarantee that a low accumulation sum (i.e., a sum that falls under the threshold) indicates that any reference sequence within the bin is a poor match with the read sequence.

This is because a lower sum means that fewer tokens from the read sequence are present in the bin, which translates directly to a greater number of errors in a potential match. For a low enough sum, we can guarantee that the potential read sequence alignment would have too many errors to be a good match.

### Taking errors into account

**Fig. 5:**
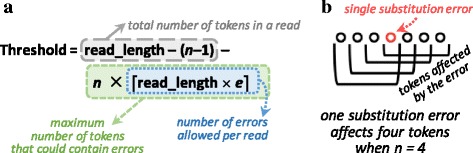
**a** Equation to calculate the accumulation sum threshold for a read sequence, where *n* is the token length and *e* is the sequence alignment error tolerance. **b** Impact of a substitution error on four separate tokens, when *n*=4. A single deletion or substitution error propagates to 4 consecutive tokens, while a single insertion error propagates to 3 consecutive tokens

If a read maps perfectly to a reference sequence in ${bin}_{bin\_num(z)}$, *Sum*_*z*_ would simply be the total number of tokens in a read, which is *read_length* − (*n*−1) for a token size of *n*. However, to account for insertions, deletions, and substitutions in the read sequence, sequence alignment has some error tolerance, where a read sequence and a reference sequence are considered a good match *even if* some differences exist. The accumulation sum threshold must account for this error tolerance, so we reduce the threshold below *read_length* − (*n*−1) to allow some tokens to include errors. Figure 5a shows the equation that we use to calculate the threshold while accounting for errors.

As shown in Fig. 5b, a token of size *n* in a bin overlaps with *n*−1 other tokens. We calculate the lowest *Sum*_*z*_ possible for a sequence alignment that includes only a single error (i.e., one insertion, deletion, or substitution) by studying these *n* tokens. If the error is an insertion, the insertion shifts at least one of the *n* tokens to the right, preserving the shifted token while changing the remaining tokens (*n*−1 in the worst case). If the error is a deletion or a substitution, the change in the worst case can affect all *n* tokens.

Figure 5b shows an example of how a substitution affects four different tokens, where *n*=4. Therefore, for each error that we tolerate, we must assume the worst-case error (i.e., a deletion or a substitution), in which case up to *n* tokens will *not* match with the read sequence even when the location actually contains the read sequence.

The equation in Fig. 5a gives the accumulation sum threshold, accounting for the worst-case scenario for a sequence alignment error tolerance of *e*. This means that the maximum number of allowable errors is equal to the *ceiling* of the read size multiplied by the sequence alignment error tolerance. A sequence alignment error tolerance of *e*=0.05 or less is widely used [2, 8, 41, 42]. For each allowable error, we assume that the worst-case number of tokens (equal to the token length *n*) are affected by the error. We also assume the worst case that each error affects a different set of tokens within the read, which results in the greatest possible number of tokens that may not match. We calculate this by multiplying the maximum number of allowable errors by *n* in the equation. Finally, we subtract the largest possible number of tokens that may *not* match from the total number of tokens in the read sequence, which is *read_length* − (*n*−1).

This leads to the threshold value that GRIM-Filter uses to determine the seed locations that the read mapper should perform sequence alignment on, as discussed in “GRIM-Filter operation” section and shown as ❺ in Fig. 3.

### Candidacy for 3D-Stacked memory implementations

We identify **three** characteristics of the filter bitmask generator in GRIM-Filter that make it a strong candidate for implementation in 3D-stacked memory: 1) it requires only very simple operations (e.g., sums and comparisons); 2) it is highly parallelizable, since each bin can be operated on independently and in parallel; and 3) it is highly memory-bound, requiring a single memory access for approximately every three computational instructions (we determine this by profiling a software implementation of GRIM-Filter, i.e., GRIM-Software, which is described in “Methods” section). Next, we describe how we implement GRIM-Filter in 3D-stacked memory.

## Mapping GRIM-Filter to 3D-stacked memory

In this section, we first describe the *3D-stacked DRAM* technology (“3D-stacked memory” section), which attempts to bridge the well-known disparity between processor speed and memory bandwidth. Next, we describe how GRIM-Filter can be easily mapped to utilize this new memory technology (“*Mapping GRIM-Filter to 3D-Stacked memory with PIM*” section). As the disparity between processor speed and memory bandwidth increases, memory becomes more of a bottleneck in the computing stack in terms of both performance and energy consumption [21, 43–46]. Along with 3D-stacked DRAM, which enables much higher bandwidth and lower latency compared to conventional DRAM, the disparity between processor and memory is alleviated by the re-emergence of the concept of *Processing-in-Memory* (PIM). PIM integrates processing units inside or near the main memory to 1) leverage high in/near-DRAM bandwidth, and low intra-DRAM latency; and 2) reduce energy consumption by reducing the amount of data transferred to and from the processor. In this section, we briefly explain the required background for these two technologies, which we leverage to implement GRIM-Filter in a highly-parallel manner.

### 3D-stacked memory

Main memory is implemented using the DRAM (dynamic random access memory) technology in today’s systems [47–49]. Conventional DRAM chips are connected to the processors using long, slow, and energy-hungry PCB (printed circuit board) interconnects [47, 49–54]. The conventional DRAM chips do not incorporate logic to perform computation. For more detail on modern DRAM operation and architecture, we refer the reader to our previous works (e.g., [47, 50–52, 54–67]).

**Fig. 6:**
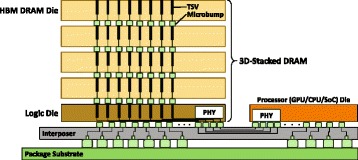
3D-stacked DRAM example. High Bandwidth Memory consists of stacked memory layers (four layers in the picture) and a logic layer connected by high bandwidth through-silicon vias (TSVs) and microbumps [13, 14, 69]. The 3D-stacked memory is then connected to a processor die with an interposer layer that provides high-bandwidth between the logic layer and the processing units on the package substrate

3D-stacked DRAM is a new DRAM technology that has a much higher internal bandwidth than conventional DRAM, thanks to the closer integration of logic and memory using the *through-silicon via* (TSV) interconnects, as seen in Fig. 6. TSVs are new, vertical interconnects that can pass through the silicon wafers of a 3D stack of dies [14, 22, 68]. A TSV has a much smaller feature size than a traditional PCB interconnect, which enables a 3D-stacked DRAM to integrate hundreds to thousands of these wired connections between stacked layers. Using this large number of wired connections, 3D-stacked DRAM can transfer bulk data simultaneously, enabling much higher bandwidth compared to conventional DRAM. Figure 6 shows a 3D-stacked DRAM (e.g., High Bandwidth Memory [13, 69]) based system that consists of four layers of DRAM dies and a logic die stacked together and connected using TSVs, a processor die, and a silicon interposer that connects the stacked DRAM and the processor. The vertical connections in the stacked DRAM are very wide and very short, which results in *high bandwidth* and *low power consumption*, respectively [14]. There are many different 3D-stacked DRAM architectures available today. High Bandwidth Memory (HBM) is already integrated into the AMD Radeon R9 Series graphics cards [15]. High Bandwidth Memory 2 (HBM2) is integrated in both the new AMD Radeon RX Vega^64^ Series graphics cards [70] and the new NVIDIA Tesla P100 GPU accelerators [71]. Hybrid Memory Cube (HMC) is developed by a number of different contributing companies [17, 18]. Like HBM, HMC also enables a logic layer underneath the DRAM layers that can perform computation [19–21]. HMC is already integrated in the SPARC64 XIfx chip [72]. Other new technologies that can enable processing-in-memory are also already prototyped in real chips, such as Micron’s Automata Processor [73] and Tibco transactional application servers [74, 75].

**Processing-in-memory (PIM).** A key technique to improve performance (both bandwidth and latency) and reduce energy consumption in the memory system is to place computation units inside the memory system, where the data resides. Today, we see processing capabilities appearing inside and near DRAM memory (e.g., in the logic layer of 3D-stacked memory) [14, 19–21, 23, 53, 76–91]. This computation inside or near DRAM significantly reduces the need to transfer data to/from the processor over the memory bus.

PIM provides significant performance improvement and energy reduction compared to the conventional system architecture [19–21, 23, 76, 90, 92, 93], which must transfer *all data* to/from the processor since the processor is the only entity that performs all computational tasks.

**3D-stacked DRAM with PIM.** The combination of the two new technologies, 3D-stacked DRAM and PIM, enables very promising opportunities to build very high-performance and low-power systems. A promising design for 3D-stacked DRAM consists of multiple stacked memory layers and a tightly-integrated logic layer that controls the stacked memory, as shown in Fig. 6. As many prior works show [14, 19–22, 76, 80, 90, 92–95], the logic layer in 3D-stacked DRAM can be utilized not only for managing the stacked memory layers, but also for integrating application-specific accelerators or simple processing cores. Since the logic layer already exists and has enough space to integrate computation units, integrating application-specific accelerators in the logic layer requires modest design and implementation overhead, and little to no hardware overhead (see [20, 89] for various analyses). Importantly, the 3D-stacked DRAM architecture enables us to fully customize the logic layer for the acceleration of applications using processing-in-memory (i.e., processing in the logic layer) [20, 21, 76, 94].

### Mapping GRIM-Filter to 3D-Stacked memory with PIM

**Fig. 7:**
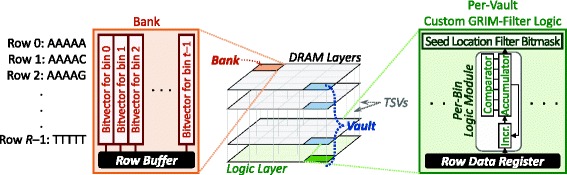
Left block: GRIM-Filter bitvector layout within a DRAM bank. Center block: 3D-stacked DRAM with tightly integrated logic layer stacked underneath with TSVs for a high intra-DRAM data transfer bandwidth. Right block: Custom GRIM-Filter logic placed in the logic layer, for each vault

We find that GRIM-Filter is a very good candidate to implement using processing-in-memory, as the filter is memory-intensive and performs simple computational operations (e.g., simple comparisons and additions). Figure 7 shows how we implement GRIM-Filter in a 3D-stacked memory. The *center block* shows each layer of an example 3D-stacked memory architecture, where multiple DRAM layers are stacked above a logic layer. The layers are connected together with several hundred TSVs, which enable a high data transfer bandwidth between the layers. Each DRAM layer is subdivided into multiple *banks* of memory. A bank in one DRAM layer is connected to banks in the other DRAM layers using the TSVs. These interconnected banks, along with a slice of the logic layer, are grouped together into a *vault*. Inside the 3D-stacked memory, we store the bitvector of each bin (see “GRIM-Filter” section) within a bank as follows: 1) each bit of the bitvector is placed in a different row in a consecutive manner (e.g., bit 0 is placed in row 0, bit 1 in row 1, and so on); and 2) all bits of the bitvector are placed in the same column, and the entire bitvector fits in the column (e.g., bitvector 0 is placed in column 0, bitvector 1 in column 1, and so on). We design and place customized logic to perform the GRIM-Filter operations within each logic layer slice, such that each vault can perform independent GRIM-Filter operations in parallel with every other vault. Next, we discuss how we organize the bitvectors within each bank. Afterwards, we discuss the customized logic required for GRIM-Filter and the associated hardware cost.

The *left block* in Fig. 7 shows the layout of bitvectors in a single bank. The bitvectors are written in column order (i.e., column-major order) to the banks, such that a DRAM access to a row fetches the existence bits of the same token across *many* bitvectors (e.g., bitvectors 0 to *t*−1 in the example in Fig. 7). When GRIM-Filter reads a row of data from a bank, the DRAM buffers the row within the bank’s *row buffer* [50, 52, 96, 97], which resides in the same DRAM layer as the bank. This data is then copied into a *row data register* that sits in the logic layer, from which the GRIM-Filter logic can read the data.

This data organization allows each vault to compute the accumulation sum of multiple bins (e.g., bins 0 to *t*−1 in the example) simultaneously. Thus, GRIM-Filter can quickly and efficiently determine, across many bins, whether a seed location needs to be discarded or sequence aligned in any of these bins.

The *right block* in Fig. 7 shows the custom hardware logic implemented for GRIM-Filter in each vault’s logic layer. We design a small *logic module* for GRIM-Filter, which consists of only an incrementer, accumulator, and comparator, and operates on the bitvector _*x*_ of a single bin *x*. The incrementer adds 1 to the value in the accumulator, which stores the accumulated sum for bin *x*. In order to hold the final sum (i.e., *Sum*_*z*_, shown as ❹ in Fig. 3), each accumulator must be at least ⌈log2(*read_length*)⌉ bits wide. Each comparator must be of the same width as the accumulator, as the comparator is used to check whether the accumulated sum exceeds the accumulated sum threshold. Because of the way we arrange the bitvectors in DRAM, a single read operation in a vault retrieves many (e.g., *t*) existence bits in parallel, from many (e.g., *t*) bitvectors, for the same token. These existence bits are copied from a DRAM bank’s row buffer into a *row data register* within the logic layer slice of the vault. In order to maximize throughput, we add a GRIM-Filter logic module *for each bin* to the logic layer slice. This allows GRIM-Filter to process all of these existence bits from multiple bitvectors in parallel.

**Integration into the system and low-level operation** When GRIM-Filter starts in the CPU (spawned by a read mapper), it sends a read sequence *r* to the in-memory GRIM-Filter logic, along with a *range* of consecutive bins to check for a match. GRIM-Filter quickly checks the range of bins to determine whether or not to discard seed locations within those bins. In the logic layer, the GRIM-Filter Filter Bitmask Generator (see “Integration with a full read mapper” section) iterates through each token in read sequence *r*. For each token, GRIM-Filter reads the memory row in each vault that contains the existence bits for that token, for the bins being checked, into the row buffer inside the DRAM layer. Then, GRIM-Filter copies the row to the row data register in the logic layer. Each GRIM-Filter logic module is assigned to a single bin. The logic module examines the bin’s existence bit in the row buffer, and the incrementer adds one to the value in the accumulator only if the existence bit is set. This process is repeated for all tokens in *r*. Once all of the tokens are processed, each logic module uses its comparator to check if the accumulator, which now holds the accumulated sum (*Sum*_*z*_, shown as ❹ in Fig. 3) for its assigned bin, is greater than or equal to the accumulated sum threshold. If *Sum*_*z*_ is greater than or equal to the threshold, a *seed location filter bit* is set, indicating that the read sequence should be sequence aligned with the locations in the bin by the read mapper. To maintain the same amount of parallelism present in the bitvector operations, we place the seed location filter bits into a *seed location filter bitmask*, where each logic module writes to one bit in the bitmask once it performs the accumulator sum threshold comparison. The seed location filter bitmask is then written to the DRAM layer. Once the Seed Location Checker (see “Integration with a full read mapper” section) starts executing in the CPU, it reads the seed location filter bitmasks from DRAM, and performs sequence alignment for only those bits whose seed location filter bits are set to 1.

**Hardware overhead.** The hardware overhead of our GRIM-Filter implementation in 3D-stacked memory depends on the available bandwidth *b* between a memory layer and the logic layer. In HBM2 [16], this bandwidth is 4096 bits per cycle across all vaults (i.e., each clock cycle, 4096 bits from a memory layer can be copied to the row data registers in the logic layer). GRIM-Filter exploits all of this parallelism completely, as we can place *b* GRIM-Filter logic modules (4096 modules for HBM2) across all vaults within the logic layer. In total, for an HBM2 memory, and for a read mapper that processes reads consisting of 100 base pairs, GRIM-Filter requires 4096 incrementer lookup tables (LUTs), 4096 seven-bit counters (a seven-bit counter can hold the maximum accumulator sum for a 100-base-pair read sequence), 4096 comparators, and enough buffer space to hold the seed location filter bitmasks. With a larger bandwidth between the logic and memory layers, we would be able to compute the seed location filter bits for more bins in parallel, but this would also incur a larger hardware overhead in the logic layer.

While the read mapper performs sequence alignment on seed locations specified by one seed location filter bitmask, GRIM-Filter generates seed location filter bitmasks for a different set of seed locations. We find that a bitmask buffer size of 512 KB (stored in DRAM) provides enough capacity to ensure that GRIM-Filter and the read mapper never stall due to a lack of buffer space.

The overall memory footprint (i.e., the amount of storage space required) of the bitvectors for a reference genome is calculated by multiplying the number of bins by the size of a single bin. In “Sensitivity to GRIM-Filter parameters” section, we show how we find a set of parameters that results in an effective filter with a low memory footprint (3.8 GB).

We conclude that GRIM-Filter requires a modest and simple logic layer, which gives it an advantage over other seed location filtering algorithms that could be implemented in the logic layer.

## Results

We first profile the reference human genome in order to 1) determine a range of parameters that are reasonable to use for GRIM-Filter. We determine the points of diminishing returns for several parameter values. This data is presented in “Sensitivity to GRIM-Filter parameters” section. Using this preliminary data, we reduce the required experiments to a reasonable range of parameters. Our implementation of GRIM-Filter enables the variation of runtime parameters (number of bins, token size, error tolerance, etc.) within the ranges of values that we determine from our experimentation for the best possible results. We then quantitatively evaluate GRIM-Filter’s improvement in false negative rate and mapper runtime over the baseline mrFAST with FastHASH (“Full mapper results” section).

### Sensitivity to GRIM-Filter parameters

In order to determine a range for the parameters for our experiments, we ran a series of analyses on the fundamental characteristics of the human reference genome. We perform these initial experiments to 1) determine effective parameters for GRIM-Filter and 2) compute its *memory footprint*. The memory footprint of GRIM-Filter depends directly on the number of bins that we divide the reference genome into, since each bin requires a bitvector to hold the token existence bits. Since the bitvector must contain a Boolean entry for each permutation of the token of size *n*, each bitvector must contain 4^*n*^ bits. The total memory footprint is then obtained by multiplying the bitvector size by the number of bins. In this section, we sweep the number of bins, token size, and error tolerance of GRIM-Filter while considering the memory footprint. To understand how each of the different parameters affect the performance of GRIM-Filter, we study a sweep on the parameters with a range of values that result in a memory footprint under 16 GB (which is the current capacity of HBM2 on state-of-the-art devices [71]).

**Fig. 8:**
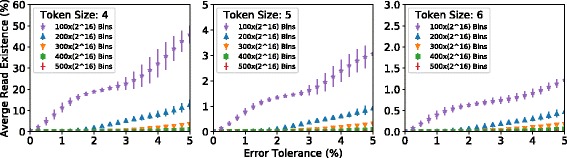
Effect of varying token size, error tolerance, and bin count on average read existence. We use a representative set of reads to collect this data. A lower value of average read existence represents a more effective filter. Note that the scale of the Y-axis is different for the three different graphs

**Average read existence** Figure 8 shows how varying a number of different parameters affects the *average read existence* across the bins. We define average read existence to be the ratio of bins with seed locations that pass the filter over all bins comprising the genome, for a representative set of reads. We would like this value to be as low as possible because it reflects the filter’s ability to filter incorrect mappings. A lower average read existence means that fewer bins must be checked when mapping the representative set of reads. Across the three plots, we vary the token size from 4 to 6. Within each plot, we vary the number of bins to split the reference genome into, denoted by the different curves (with different colors and markers). The X-axis shows the error tolerance that is used, and the Y-axis shows average read existence. We plot the average and min/max across our 10 data sets (Table 1) as indicated, respectively, by the triangle and whiskers. We sweep the number of bins in multiples of 2^16^ because 2^16^ is an even multiple of the number of TSVs between the logic and memory layers in today’s 3D-stacked memories (today’s systems typically have 4096 TSVs). We want to use a multiple of 2^16^ so that we can utilize all TSVs each time we copy data from a row buffer in the memory layer to the corresponding row data register in the logic layer. This maximizes GRIM-Filter’s internal memory bandwidth utilization within 3D-stacked memory.

**Table 1:** Benchmark data, obtained from the 1000 Genomes Project [102]

	ERR240726_1	ERR240727_1	ERR240728_1	ERR240729_1	ERR240730_1
No. of reads	4031354	4082203	3894290	4013341	4082472
Read length	100	100	100	100	100
	ERR240726_2	ERR240727_2	ERR240728_2	ERR240729_2	ERR240730_2
No. of reads	4389429	4013341	4013341	4082472	4082472
Read length	100	100	100	100	100

We make *three* observations from the figure. First, looking across the three plots, we observe that increasing the *token size* from 4 to 5 provides a large (i.e., around 10x) reduction in average read existence, while increasing the token size from 5 to 6 provides a much smaller (i.e., around 2x) reduction in average read existence. The reduction in average read existence is due to the fact that, in a random pool of As, Cs, Ts, and Gs, the probability of observing a certain substring of size *q* is $\left ({\frac {1}{4}}\right)^{q}$. Because the distribution of base pairs across a reference genome and across a bin is *not* random, a larger token size does *not* always result in a large decrease, as seen when changing the token size from 5 to 6. We note that increasing the token size by one causes GRIM-Filter to use 4x the memory footprint. Second, we observe that in all three plots (i.e., for all token sizes), an increase in the number of bins results in a decrease in the average read existence. This is because the bin size decreases as the number of bins increases, and for smaller bins, we have a smaller sample size of the reference genome that any given substring could exist within. Third, we observe that for each plot, increasing the error tolerance results in an increase in the average read existence. This is due to the fact that if we allow more errors, fewer tokens of the entire read sequence must be present in a bin for a seed location from that bin to pass the filter. This increases the probability that a seed location of a random read passes the filter for a random bin. A poor sequence alignment at a location that passes the filter is categorized as a false negative. We conclude from this figure that using tokens of size 5 provides quite good filtering effectiveness (as measured by average read existence) without requiring as much memory footprint as using a token size of 6.

**Fig. 9:**
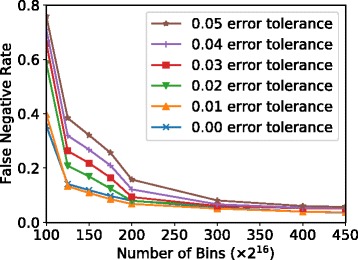
GRIM-Filter’s false negative rate (lower is better) as we vary the number of bins. We find that increasing the number of bins beyond 300×2^16^ yields diminishing improvements in the false negative rate, regardless of the error tolerance value

**False negative rate.** We choose our final bitvector size after sweeping the number of bins and the error tolerance (*e*). Figure 9 shows how varying these parameters affects the false negative rate of GRIM-Filter. The X-axis varies the number of bins, while the different lines represent different values of *e*.

We make two observations from this figure. First, we find that, with more bins (i.e., with a smaller bin size), the false negative rate (i.e., the fraction of locations that pass the filter, but do not result in a mapping after alignment) decays exponentially. Above 300 ×2^16^ bins, we begin to see diminishing returns on the reduction in false negatives for all error tolerance values. Second, we observe that, as we increase the error tolerance, regardless of the other parameters, the false negative rate increases. We also find that the number of bins 1) minimally affects the runtime of GRIM-Filter (not plotted) and 2) linearly increases the memory footprint. Based on this study, we choose to use 450 ×2^16^ bins, which reflects a reasonable memory footprint (see below) with the other parameters.

**Memory footprint.** A larger number of bins results in more bitvectors, so we must keep this parameter at a reasonable value in order to retain a reasonable memory footprint for GRIM-Filter. Since we have chosen a token size of 5, GRIM-Filter requires *t* bitvectors with a length of 4^5^=1024, where *t* equals the number of bins we segment the reference genome into. We conclude that employing 450 ×2^16^ bins results in the best trade-off between memory footprint, filtering efficiency, and runtime. This set of parameters results in a total memory footprint of approximately 3.8 GB for storing the bitvectors of this mechanism, which is a very reasonable size for today’s 3D-stacked memories [13, 15, 17, 18, 69–71]. We note that the time to generate the bitvectors is *not* included in our final runtime results, because these need to be generated only once per reference genome, either by the user or by the distributor. We find that, with a genome of length *L*, we can generate the bitvectors in (9.03*e*−08)×*L* seconds when we use 450 ×2^16^ bins (this is approximately 5 min for the human genome).

**GRIM-Filter parallelization.** GRIM-Filter operates on every bin independently and in parallel, using a separate logic module for each bin. Thus, GRIM-Filter’s parallelism increases with each additional bin it operates on simultaneously. We refer to the set of consecutive bins that the GRIM-Filter logic modules are currently assigned to as the *bin window* (*w*). The internal bandwidth of HBM2 [16] enables copying 4096 bits from a memory layer to the logic layer every cycle, allowing GRIM-Filter to operate on as many as 4096 consecutive bins in parallel (i.e., it has a bin window of size *w*=4096). GRIM-Filter must only check bin windows that contain at least one seed location (i.e., a span of 4096 consecutive bins with *zero* seed locations does not need to be checked). In contrast, if a consecutive set of 4096 bins contains many seed locations, GRIM-Filter can operate on every bin in parallel and quickly determine which seed locations within the 4096 bins can safely be discarded. In these cases, GRIM-Filter can most effectively utilize the parallelism available from the 4096 independent logic modules.

In order to understand GRIM-Filter’s ability to parallelize operations on many bins, we analyze GRIM-Filter when using a bin window of size *w*=4096, which takes advantage of the full memory bandwidth available in HBM2 memory. As we discuss in “Integration with a full read mapper” section, the read mapper generates a list of potential seed locations for a read sequence, and sends this list to GRIM-Filter when the filter starts. Several bins, which we call *empty bins*, do not contain *any* potential seed locations. When *w*=1, there is only one logic module, and if the module is assigned to an empty bin, GRIM-Filter immediately moves on to the next bin without computing the accumulation sum. However, when *w*=4096, some, but not all, of the logic modules may be assigned to empty bins. This happens because in order to simplify the hardware, GRIM-Filter operates all of the logic modules in lockstep (i.e., the filter fetches a *single row* from each bank of memory, which includes the existence bits *for a single token* across multiple rows, and all of the logic modules read and process the existence bits for the same token in the same cycle). Thus, a logic module assigned to an empty bin must wait for the other logic modules to finish before it can move onto another bin. As a result, GRIM-Filter with *w*=4096 is not 4096x faster than GRIM-Filter with *w*=1. To quantify the benefits of parallelization, we compare the performance of GRIM-Filter with these two bin window sizes using a representative set of reads. For 10% of the seeds, we find that GRIM-Filter with *w*=4096 reduces the filtering time by 98.6%, compared to GRIM-Filter with *w*=1. For the remaining seeds, we find that GRIM-Filter with *w*=4096 reduces the filtering time by 10–20%. Thus, even though many of the logic modules are assigned to empty bins in a given cycle, GRIM-Filter reduces the filtering time by operating on many bins that contain potential seed locations in parallel.

**Overlapping GRIM-Filter computation with sequence alignment in the CPU.** In addition to operating on multiple bins in parallel, one benefit of implementing GRIM-Filter in 3D-stacked memory is that filtering operations can be parallelized with sequence alignment that happens on the CPU, since filtering no longer uses the CPU. Every cycle, for a bin window of size *w*=4096, GRIM-Filter’s Filter Bitmask Generator (❶ in Fig. 4) reads 4096 bits from memory, and updates the accumulation sums for the bins within the bin window that contain a potential seed location. Once the accumulation sums are computed and compared against the threshold, GRIM-Filter’s Seed Location Checker (❷ in Fig. 4) can discard seed locations that map to bins whose accumulation sums do *not* meet the threshold (i.e., the seed locations that should not be sent to sequence alignment). The seed locations that are not discarded are sent to the read mapper for sequence alignment (❹ in Fig. 4), ending GRIM-Filter’s work for the current bin window. While the read mapper aligns the sequences that passed through the filter from the completed bin window, GRIM-Filter’s Filter Bitmask Generator moves onto another bin window, computing the seed location filter bits for a new set of bins. If GRIM-Filter can exploit enough parallelism, it can provide the CPU with enough bins to keep the sequence alignment step busy for at least as long as the time needed for the Filter Bitmask Generator to process the new bin window. This would allow the filtering latency to overlap *completely* with alignment, in effect hiding GRIM-Filter’s latency. We find that a bin window of 4096 bins provides enough parallelism to *completely* hide the filtering latency while the read mapper running on the CPU performs sequence alignment.

### Full mapper results

We use a popular seed-and-extend mapper, mrFAST [3], to retrieve all candidate mappings from the ten real data sets we evaluate (see “Methods” section). In our experiments, we use a token size of 5 and 450×2^16^ bins, as discussed in *Sensitivity to GRIM-Filter Parameters*. All remaining parameters specific to mrFAST are held at the default values across all of our evaluated read mappers.

**Fig. 10:**
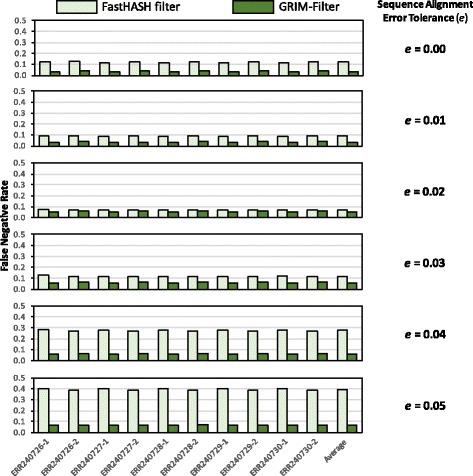
False negative rates of GRIM-Filter and FastHASH filter across ten real data sets for six different error tolerance values

**False negative rate.** Figure 10 shows the false negative rate of GRIM-Filter compared to the baseline FastHASH filter across the ten real data sets we evaluate. The six plots in the figure show false negative rates for error tolerance values (i.e., *e*) ranging from 0.00 to 0.05, in increments of 0.01 (An error tolerance of *e*=0.05 is widely used in alignment during DNA read mapping [2, 8, 41, 42].). We make three observations from the figure. First, GRIM-Filter provides a much lower false negative rate than the baseline FastHASH filter for all data sets and for all error tolerance values. For an error tolerance of *e*=0.05 (shown in the bottom graph), the false negative rate for GRIM-Filter is *5.97x* lower than for FastHASH filter, averaged across all 10 read data sets. Second, GRIM-Filter’s false negative rate 1) increases as the error tolerance increases from *e*=0.00 to *e*=0.02, and then 2) decreases as the error tolerance increases further from *e*=0.03 to *e*=0.05. There are at least two conflicting reasons. First, as the error tolerance increases, the accumulation sum threshold decreases (as shown in Fig. 5) and thus GRIM-Filter discards *fewer* locations, which results in a *higher* false negative rate. Second, as the error tolerance increases, the number of acceptable (i.e., correct) mapping locations increases while the number of candidate locations remains the same, which results in a *lower* false negative rate. The interaction of these conflicting reasons results in the initial increase and the subsequent decrease in the false negative rates that we observe. Third, we observe that for higher error tolerance values, GRIM-Filter reduces the false negative rate compared to the FastHASH filter by a larger fraction. This shows that GRIM-Filter is much more effective at filtering mapping locations when we increase the error tolerance. We conclude that GRIM-Filter is very effective in reducing the false negative rate.

**Fig. 11:**
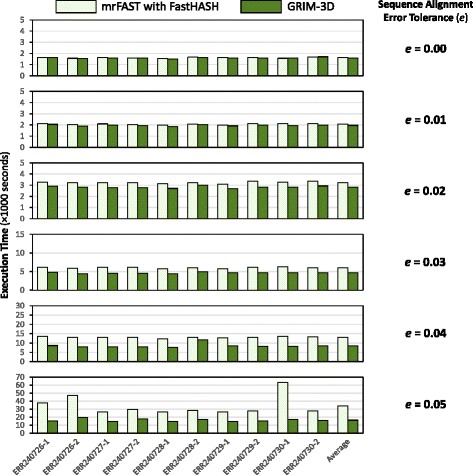
Execution time of two mappers, GRIM-3D and mrFAST with FastHASH, across ten real data sets for six different error tolerance values Note that the scale of the Y-axis is different for the six different graphs

**Execution time.** Figure 11 compares the execution time of GRIM-3D to that of mrFAST with FastHASH across all ten different read data sets for the same error tolerance values used in Fig. 10. We make three observations. First, GRIM-3D improves performance for all of our data sets for all error tolerance values. For an error tolerance of *e*=0.05, the average (maximum) performance improvement is 2.08x (3.65x) across all 10 data sets. Second, as the error tolerance increases, GRIM-3D’s performance improvement also increases. This is because GRIM-Filter safely discards many more mapping locations than the FastHASH filter at higher error tolerance values (as we showed in Fig. 10). Thus, GRIM-Filter saves significantly more execution time than the FastHASH filter by ignoring many more unnecessary alignments. Third, based on an analysis of the execution time breakdown of GRIM-3D (not shown), we find that GRIM-3D’s performance gains are mainly due to an 83.7% reduction in the average computation time spent on false negatives, compared to using the FastHASH filter for seed location filtering. We conclude that employing GRIM-Filter for seed location filtering in a state-of-the-art read mapper significantly improves the performance of the read mapper.

## Related work

To our knowledge, this is the first paper to exploit 3D-stacked DRAM and its processing-in-memory capabilities to implement a new seed location filtering algorithm that mitigates the major bottleneck in read mapping, pre-alignment (i.e., seed location filtering). In this section, we briefly describe related works that aim to 1) accelerate pre-alignment algorithms, and 2) accelerate sequence alignment with hardware support.

**Accelerating Pre-Alignment.** A very recent prior work [9] implements a seed location filter in an FPGA, and shows significant speedup against prior filters. However, as shown in that work, the FPGA is still limited by the memory bandwidth bottleneck. GRIM-Filter can overcome this bottleneck on an FPGA as well.

**Accelerating sequence alignment.** Another very recent prior work [98] exploits the high memory bandwidth and the reconfigurable logic layer of 3D-stacked memory to implement an accelerator for sequence alignment (among other basic algorithms within the sequence analysis pipeline). Many prior works (e.g., [26–36]) use FPGAs to also accelerate sequence alignment. These works accelerate sequence alignment using customized FPGA implementations of different existing read mapping algorithms. For example, Arram et al. [28] accelerate the SOAP3 tool on an FPGA engine, achieving up to 134x speedup compared to BWA [99]. Houtgast et al. [32] present an FPGA-accelerated version of BWA-MEM that is 3x faster compared to its software implementation. Other works use GPUs [37–40] for the same purpose of accelerating sequence alignment. For example, Liu et al. [38] accelerate BWA and Bowtie by 7.5x and 20x, respectively. In contrast to GRIM-Filter, all of these accelerators focus on accelerating sequence alignment, whereas GRIM-Filter accelerates pre-alignment (i.e., seed location filtering). Hence, GRIM-Filter is orthogonal to these works, and can be combined with any of them for further performance improvement.

## Discussion

We have shown that GRIM-Filter significantly reduces the execution time of read mappers by reducing the number of unnecessary sequence alignments and by taking advantage of processing-in-memory using 3D-stacked DRAM technology. We believe there are many other possible applications for employing 3D-stacked DRAM technology within the genome sequence analysis pipeline (as initially explored in [98]), and significant additional performance improvements can be obtained by combining future techniques with GRIM-Filter. Because GRIM-Filter is essentially a seed location filter to be employed before sequence alignment during read mapping, it can be used in any other read mapper along with any other acceleration mechanisms in the genome sequence analysis pipeline.

We identify three promising major future research directions. We believe it is promising to 1) explore the benefits of combining GRIM-Filter with other various read mappers in the field, 2) show the effects of mapping to varying sizes of reference genomes, and 3) examine how GRIM-Filter can scale to process a greater number of reads concurrently.

## Conclusion

This paper introduces GRIM-Filter, a novel algorithm for seed location filtering, which is a critical performance bottleneck in genome read mapping. GRIM-Filter has three major novel aspects. First, it preprocesses the reference genome to collect metadata on large subsequences (i.e., *bins*) of the genome and stores information on whether small subsequences (i.e., *tokens*) are present in each bin. Second, GRIM-Filter efficiently operates on the metadata to quickly determine whether to discard a mapping location for a read sequence prior to an expensive sequence alignment, thereby reducing the number of unnecessary alignments and improving performance. Third, GRIM-Filter takes advantage of the logic layer within 3D-stacked memory, which enables the efficient use of processing-in-memory to overcome the memory bandwidth bottleneck in seed location filtering. We examine the trade-offs for various parameters in GRIM-Filter, and present a set of parameters that result in significant performance improvement over the state-of-the-art seed location filter, FastHASH. When running with a sequence alignment error tolerance of 0.05, we show that GRIM-Filter 1) filters seed locations with *5.59x–6.41x* lower false negative rates than FastHASH; and 2) improves the performance of the fastest read mapper, mrFAST with FastHASH, by *1.81x–3.65x*. GRIM-Filter is a universal seed location filter that can be applied to any read mapper.

We believe there is a very promising potential in designing DNA read mapping algorithms for new memory technologies (like 3D-stacked DRAM) and new processing paradigms (like processing-in-memory). We hope that the results from our paper provide inspiration for other works to design new sequence analysis and other bioinformatics algorithms that take advantage of new memory technologies and new processing paradigms, such as processing-in-memory using 3D-stacked DRAM.

## Methods

**Evaluated read mappers.** We evaluate our proposal by incorporating GRIM-Filter into the state-of-the-art hash table based read mapper, mrFAST with FastHASH [7]. We choose this mapper for our evaluations as it provides high accuracy in the presence of relatively many errors, which is required to detect genomic variants within and across species [3, 7]. GRIM-Filter plugs in as an extension to mrFAST, using a simple series of calls to an application programming interface (API). However, we note that GRIM-Filter can be used with any other read mapper.

We evaluate two read mappers:

- *mrFAST with FastHASH* [7], which does *not* use GRIM-Filter;
- *GRIM-3D*, our 3D-stacked memory implementation of GRIM-Filter combined with mrFAST and the non-filtering portions of FastHASH.

**Major evaluation metrics.** We report 1) GRIM-Filter’s *false negative rate* (i.e., the fraction of locations that pass through the filter but do not contain a match with the read sequence), and 2) the end-to-end *performance improvement* of the read mapper when using GRIM-Filter.

We measure the false negative rate of our filter (and the baseline filter used by the mapper) as the ratio of the number of locations that passed the filter but did not result in a mapping over all locations that passed the filter. Note that our implementation of GRIM-Filter ensures a zero *false positive rate* (i.e., it does *not* filter out any correct mappings for the read sequence), and, thus, GRIM-Filter does not affect the correctness of a read mapper.

**Performance evaluation.** We measure the performance improvement of GRIM-3D by comparing the execution time of our read mappers. We develop a methodology to *estimate* the performance of GRIM-3D, since real hardware systems that enable in-memory computation are unavailable to us at this point in time. To estimate GRIM-3D’s execution time, we need to add up the time spent by three components (which we denote as *t*_*x*_ for component *x*):

- *t*_1_: the time spent on read mapping,
- *t*_2_: the time spent on coordinating which bins are examined by GRIM-Filter, and
- *t*_3_: the time spent on applying the filter to each seed.

To obtain *t*_1_ and *t*_2_, we measure the performance of *GRIM-Software*, a software-only version of GRIM-Filter that does *not* take advantage of processing in 3D-stacked memory. We run GRIM-Software with mrFAST, and measure:

- *GRIM-Software-End-to-End-Time*, the end-to-end execution time for read mapping using GRIM-Software;
- *GRIM-Software-Filtering-Time*, the time spent only on applying the filter (i.e., the GRIM-Filter portions of the code shown in Fig. 4) using GRIM-Software.

The values of *t*_1_ and *t*_2_ are the same for GRIM-Software and GRIM-3D, and we can compute those by subtracting out the time spent on filtering from the end-to-end execution time: *t*_1_+*t*_2_=GRIM-Software-End-to-End-Time−GRIM-Software-Filtering-Time. To estimate *t*_3_, we use a validated simulator similar to Ramulator [47, 100], which provides us with the time spent by GRIM-3D on filtering using processing-in-memory. The simulator models the time spent by the in-memory logic to produce a seed location filter bitmask, and to store the bitmask into a buffer that is accessible by the read mapper.

**Evaluation system.** We evaluate the software versions of the read mappers (i.e., mrFAST with FastHASH and GRIM-Software) using an Intel(R) Core i7-2600 CPU running at 3.40 GHz [101], with 16 GB of DRAM for all experiments.

**Data sets.** We used ten real data sets from the 1000 Genomes Project [102]. We used the same data sets used by Xin et al. [7] for the original evaluation of mrFAST with FastHASH, in order to provide a fair comparison to our baseline. Table 1 lists the read length and size of each data set.

**Code availability.** The code for GRIM-Filter, GRIM-Software, and our simulator for 3D-stacked DRAM with processing-in-memory is freely available at https://github.com/CMU-SAFARI/GRIM.
